# Scalar Relativistic Effects with Multiwavelets: Implementation and Benchmark

**DOI:** 10.1021/acs.jctc.3c01095

**Published:** 2024-01-05

**Authors:** Anders Brakestad, Stig Rune Jensen, Christian Tantardini, Quentin Pitteloud, Peter Wind, Jānis Užulis, Andris Gulans, Kathrin Helen Hopmann, Luca Frediani

**Affiliations:** † Hylleraas Centre for Quantum Molecular Sciences, UiT The Arctic University of Norway, Tromsø 9037, Norway; ‡ Department of Chemistry, UiT The Arctic University of Norway, Tromsø 9037, Norway; § Department of Materials Science and NanoEngineering, Rice University, Houston, Texas 77005, United States; ∥ Department of Physics, University of Latvia, Jelgavas iela 3, Riga, Latvia 1004, Latvia

## Abstract

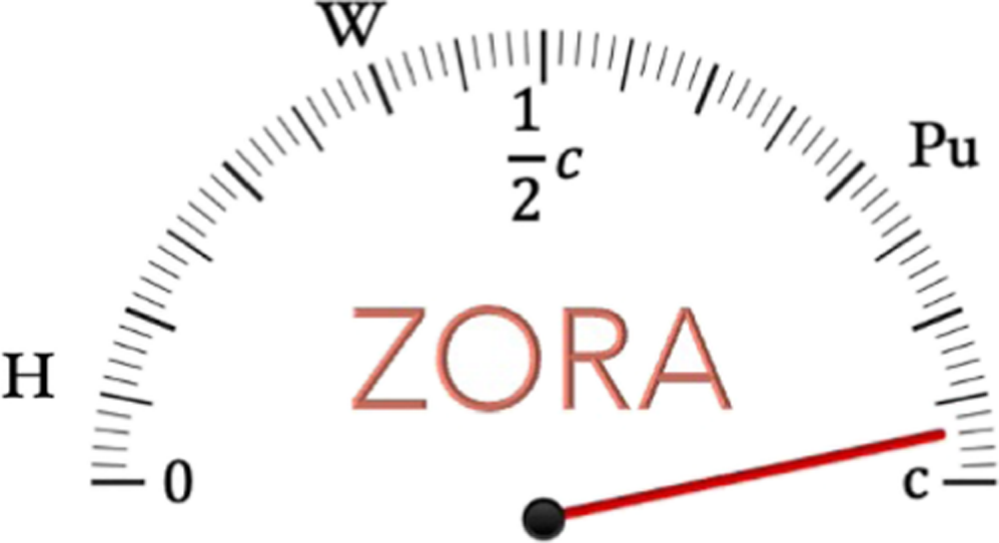

The importance of relativistic effects in quantum chemistry is widely recognized, not only for heavier elements but throughout the periodic table. At the same time, relativistic effects are strongest in the nuclear region, where the description of electrons through a linear combination of atomic orbitals becomes more challenging. Furthermore, the choice of basis sets for heavier elements is limited compared with lighter elements where precise basis sets are available. Thanks to the framework of multiresolution analysis, multiwavelets provide an appealing alternative to overcoming this challenge: they lead to robust error control and adaptive algorithms that automatically refine the basis set description until the desired precision is reached. This allows one to achieve a proper description of the nuclear region. In this work, we extended the multiwavelet-based code MRChem to the scalar zero-order regular approximation framework. We validated our implementation by comparing the total energies for a small set of elements and molecules. To confirm the validity of our implementation, we compared both against a radial numerical code for atoms and the plane-wave-based code EXCITING.

## Introduction

In his famous Nobel Price Lecture, P.A.M. Dirac stated that with the advent of quantum mechanics, all fundamental problems of chemistry were in principle solved.^1^ It is however interesting that he did not seem to realize that relativity would also play a role, despite his own fundamental contribution to combining relativity and quantum mechanics.^2,3^ It is now widely accepted that the correct description of the electronic structures of atoms and molecules requires the inclusion of relativistic effects. This is particularly relevant for the core- and the innermost valence shell-electrons of heavier elements, which move at sizable fractions of the speed of light. A wide number of chemical properties and phenomena, such as the yellow color of gold,^4,5^ the liquid state of mercury,^5,6^ the functioning of lead-acid batteries,^5,7^ the catalytic behavior of cobalt,^5,8−10^ and the difficulties to describe fluorinated compounds with density functional theory (DFT),^11−13^ all are due to relativistic effects. There is a wide spectrum of methods that include relativistic effects in electronic structure calculations. They range from the use of effective core potential (ECP),^14−17^ also known as pseudopotentials in the solid-state physics community,^18^ to the solution of the four-component Dirac equation. In between these two extremes, there are a number of methods with different degrees of accuracy and complexity.^19^

The ECP method collapses a given number of core electrons and the nucleus to provide a core potential for the valence electrons. This has the dual advantage of reducing the number of explicit electrons while at the same time implicitly including relativistic effects. On the other hand, if core electronic properties such as high pressure chemistry, core–electron spectroscopy^20^ and nuclear magnetic resonance (NMR) shielding constants^9^ are considered, ECP methods fall short, and all-electron calculations are necessary. The four-component Dirac equation^2^ constitutes the starting point for the treatment of relativistic effects, and the full Breit Hamiltonian^21−25^ is the most complete treatment of relativistic effects for a many-electron system. On the other hand, it is also the most computationally expensive: spin–orbitals are four-component complex functions. Spin is no longer a good quantum number in a relativistic framework, and the simplified picture of two electrons with opposite spin sharing the same orbital is no longer valid.^19,26,27^ Most operators couple the different components of a spin–orbital, leading roughly to a factor 100 in computational cost, because coupling four complex functions requires 8 × 8 matrices instead of a real scalar.^28−31^ Although progress has been made to make four-component calculations faster and easily available,^28−31^ it is still convenient to attempt approximations that promise great reduction in the computational cost at the price of reduced accuracy. The first step in such a hierarchy of approximations is the elimination of the small components through a Foldy–Wouthuysens (FWs) transformation.^32,33^ The choice of transformation leads to different kinds of methods, such as the Pauli Hamiltonian,^34−36^ the regular approximations (RAs),^37−39^ the Douglas–Kroll–Hess (DKH) Hamiltonian,^40−44^ or the exact two-component (X2C) Hamiltonian method.^19,45−53^ Further reduction to a scalar method is also possible for the Pauli Hamiltonian and the RAs.^37−39^ In particular, the RAs have two interesting features: (1) the decoupling part of the FWs transformation can be expanded in a convergent series to recover the exact elimination, and (2) the renormalization part can be exactly incorporated into the wave function. The zero-order regular approximation (ZORA)^34,37,54−56^ is the simplest form of Hamiltonian keeping only the zeroth order in both parts. The reduction to a scalar method is carried out by applying the Dirac identity and discarding the spin–orbit term. The advantage of ZORA is to keep most of the standard algorithms of quantum chemistry in their original form just rescaling the kinetic energy by a function that includes the potential energy (see below Theory and Implementation section).^34,37,54−56^ The numerical treatment of this rescaling function can be challenging because it displays a cusp at each nucleus.^34,37,54−56^ Standard approaches, based on atomic orbital expansions, can struggle to obtain an accurate description in this region. The issue is further aggravated for heavier nuclei, where such a correction is important, and at the same time, the number of basis sets available is more limited and less is known about their true precision.^57,58^

Some efforts to assess the precision of ZORA for all-electron calculations of heavier elements have been undertaken using Gaussian type orbitals (GTOs), but it is anyway challenging to assess the precision without an external reference.^57,58^ One such option for hydrogen-like ions (He^+^, Ne^9+^, Ar^17+^, etc.) is constituted by the scaling properties of the ZORA Hamiltonian in a two-component framework, which yields the (scaled) exact Dirac energies for such a system.^59^ However, for many-electron systems and/or for a scalar relativistic approach, assessing the true precision of a GTO basis is challenging, and several basis sets are developed to include relativistic effects. Examples of all-electron relativistic basis sets include the universal Gaussian basis set (UGBS),^60^ the atomic natural orbitals basis sets,^61−65^ the X2C basis sets,^66^ and the segmented all-electron relativistic contracted basis sets.^67−71^ Importantly, such basis sets must be fitted to the chosen Hamiltonian (ZORA and DKH). This comes on top of the required fitting of the basis set to a given electronic structure method, and if relevant, to a particular property.^71^ Although some of the known all-electron relativistic basis sets may provide good results for a particular method and property, their transferability is limited. The large number of available GTO basis sets is an indication that no single basis set is good enough to describe all properties of interest to sufficient precision.^58^

In recent years, multiwavelets (MWs)^72^ have emerged as a powerful alternative to traditional local basis sets.^73^ Their foundation based on multiresolution analysis^74^ leads to a basis set that is not empirically parametrized. Robust error control^75−77^ means that the user can set a finite but arbitrary target precision, and adaptive algorithms^78−80^ ensure that the representation of molecular orbitals is automatically refined until the required precision is reached. Instead of a plethora of bases to choose from, the user only needs to set the requested precision and the polynomial order of the basis, providing in practice a robust black-box, where straightforward numerical considerations guide the user’s choice. MWs have proven reliable and robust in providing Hartree–Fock (HF) and DFT benchmark results for energies^73^ and properties^81^ and have ventured both toward post-HF methods^82^ and relativistic treatments.^83^

In this work, we present a MW implementation of the ZORA method in the MRChem code. To verify the correctness of the implementation, we consider two alternative methods: a radial atomic solver implemented as a separate package^84^ and linearized augmented plane wave (LAPW) implemented in the electronic-structure code EXCITING.^85,104^ Both of these approaches provide means for systematic improvement of the precision and are capable of yielding total energies approaching the complete basis set limit, as demonstrated in refs (84) and (86).

## Theory and Implementation

### From the Dirac Equation to the ZORA Hamiltonian

We will briefly expose how the ZORA^34,37,54−56^ is derived starting from the four-component Dirac equation of an electron, which describes a four-component spinor Ψ, subject to a potential *V*
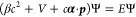
1

In the above equation ***p*** is the momentum operator, *c* is the speed of light, atomic units (*m*_*e*_ = 1, *ℏ* = 1, *e* = −1) are assumed and **α** and β are defined as follows
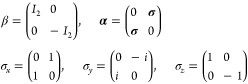
2

The first step toward ZORA consists in applying the FW transformation to the Dirac Hamiltonian of eq 1
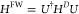
3where the transformation matrix *U* = *W*_1_*W*_2_ is a product of a decoupling matrix *W*_1_ and a renormalization matrix *W*_2_
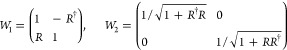
4and *R* is the exact coupling between the large and the small components of a four-spin–orbits
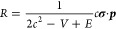
5

The inverse potential term depends on the eigenvalue *E*, and this dependence can be expanded in a Taylor series as follows
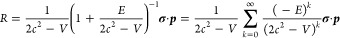
6

Restricting the expansion to the zeroth order leads to RA. Once the renormalization *W*_2_ is also considered, the following two-component Hamiltonian is obtained
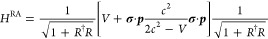
7

The ZORA Hamiltonian is finally obtained by Taylor-expanding the renormalization operator and retaining only the zero-order term
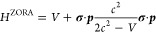
8

Using the Dirac identity
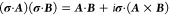
9we can separate the scalar-relativistic and spin–orbit contributions to the kinetic energy covered by the first and the second terms, respectively. Here, we keep only the scalar-relativistic part and obtain the following Hamiltonian
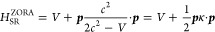
10where in the last expression, we have implicitly defined . Given the ZORA Hamiltonian, a Kohn–Sham (KS) DFT implementation is then obtained by replacing the nonrelativistic kinetic energy operator with its ZORA counterpart
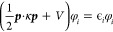
11

It should be noted that in practical implementations, the potential defining κ is usually *not* the full KS potential. Given the form of κ, the most important contribution is nuclear attraction. Introducing Coulombic  and exchange and correlation  is possible, but the corresponding operator has to be recomputed numerically at each iteration. Additionally, operations such as function multiplications are difficult to perform in traditional linear combination atomic orbital basis representations. A common choice for GTO calculations is to use a fixed atomic potential, which is called the atomic-ZORA approximation.^87^ The ZORA kinetic operator can then be precomputed and used throughout the calculation. Atomic-ZORA has the advantage of being gauge-invariant.^87^

### ZORA Equations in a Multiwavelet Framework

To obtain a MW implementation of the ZORA eigenvalue problem, it is necessary to transform the differential equation into an integral equation, in analogy with the nonrelativistic case.^75,77^ The standard KS equations can be concisely written as follows
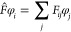
12where *F̂* is the Fock operator, φ_*i*_ refer to an occupied molecular orbital, and *F*_*ij*_ are the matrix elements of the Fock operator between two occupied orbitals, assuming a general noncanonical (nondiagonal) form.

Within the framework of KS-DFT, the Fock operator consists of the kinetic energy *T̂*, the nuclear attraction , the Coulombic repulsion *Ĵ*, the HF exchange *K̂* scaled by some numerical factor λ ∈ [0, 1], and the exchange and correlation potential 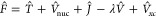
13

In the nonrelativistic domain, the coupled KS differential (eq 12) can be rewritten in the integral form,^88,89^ by making use of the bound-state Helmholtz kernel
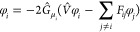
14where  is the integral convolution operator associated with the bound-state Helmholtz kernel , using  (i.e., the diagonal elements of the Fock matrix).

In the ZORA Hamiltonian, the kinetic energy operator becomes
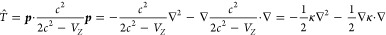
15where . Including *Ĵ* and  does not pose any issue in the MW framework, other than the computational overhead of having to update the potential at every iteration, because all potentials are anyway treated on an equal footing using a numerical grid. The nonlocal HF exchange, in turn, does not seem to contribute significantly to *V*_*Z*_, as shown in ref (90).

Inserting the ZORA kinetic operator into eq 12, we obtain
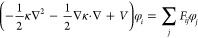
16

In order to make use of the same framework as the nonrelativistic implementation of eq 14, it is necessary to isolate the Laplacian and the diagonal element of the sum on the right-hand side, which together make up the bound-state Helmholtz operator . This is achieved first by division by κ, recalling that κ^–1^ = 1 – *V*_*Z*_/2*c*. The following integral equation is obtained
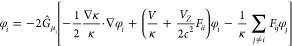
17

When *c* → *∞*, κ → 1, and ∇κ → 0, and the nonrelativistic form as in eq 14 is recovered. Equation 17 can therefore be seen as a *level-shifted* version of its nonrelativistic counterpart. Although it cannot be expected that the iterative solution of eq 17 will work for arbitrary shifts, the approach is justified by recalling that κ ≃ 1 almost everywhere except close to the nuclei. Our tests indicate that it becomes more difficult to converge the above equation when the ZORA contribution becomes larger, either *physically* by going down the periodic table, or *artificially* by letting *c* → 0. To overcome the convergence issues for heavier nuclei (fifth row of the periodic table), we have therefore introduced a finite nucleus model, as described in the section Finite Nucleus Model. For elements beyond the fifth row, one has to keep in mind that ZORA becomes questionable.^91^

An alternative approach to obtain the desired (∇^2^ + 2*F*_*ii*_) term from eq 16 is to add a Laplacian term  directly, thus avoiding division by κ. Our tests indicate that the strategy presented above works better, despite the additional singularity introduced. The main reason seems to be that the former strategy removes the Laplacian altogether, which is ill-conditioned in the discontinuous MW basis, whereas the latter keeps part of it on the right-hand side.

### Implementation within Multiwavelets

An implementation of the ZORA method as outlined above is currently in a development version of the MRChem package^92^ and is expected to appear in the next official release v1.2. MRChem is a numerical quantum chemistry code based on a MW framework, in which all functions and operators are represented on their own fully adaptive multiresolution numerical real-space grid. This allows for efficient all-electron treatment of medium to large molecules (hundreds of atoms) at the self-consistent field (SCF) level of theory (both HF and DFT).

The κ function is computed as a pointwise map of the chosen ZORA potential *V*_*Z*_ through
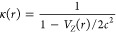
18and similarly for its inverse
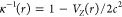
19

Both functions are represented on their own adaptive numerical grid and subsequently treated as standard multiplicative potential operators in the SCF procedure of solving eq 17.

### Point Nucleus Model

In a MW framework, the singularity of the nuclear potential can lead to numerical problems when *V*_nuc_ is computed and used. In the nonrelativistic case, the issue is circumvented by replacing the analytic 1/*r* potential with a smoothed approximation^75^
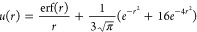
20and then parametrized as *u*(*r*/*s*)/*s*, where *s* is a scalar smoothing parameter. The smoothing parameter depends on the nuclear charge *Z* and the desired precision ϵ as explained in ref (75).
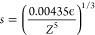
21

The above prescription constitutes a *numerical* smoothing of the nucleus to avoid accidental infinity in the representations. It should not be confused with *physical* finite nucleus models,^93^ which are common in relativistic methods. This numerical smoothing is much sharper than the common finite nucleus models, and it is meant to yield results that are, within the requested precision, equivalent to using a point charge.

### Finite Nucleus Model

In order to overcome the numerical issues faced by the pointwise nuclei, we have introduced the Gaussian nuclear model, as described by Visscher and Dyall.^93^ Not only does the model overcome the numerical problems of pointwise charges but it is also a sounder physical description of larger nuclei. The nuclear charge is modeled as a Gaussian distribution
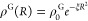
22with the normalization prefactor ρ_0_^G^ = *eZ*(ξ/π)^3/2^ and the parameter ξ related to the root-mean-square radius of the nucleus via the expression ξ = 3/⟨*R*^2^⟩. To determine ⟨*R*^2^⟩, we apply the empirical formula for ref (94)
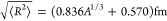
23where *A* is the nuclear mass number and fm is a femtometer length unit (10^–15^ m). We note that a choice of the nuclear mass *A* is dependent on whether the abundance of isotopes is taken in to account. To avoid the ambiguity, we use the tabulated values of  provided in ref (93).

The potential for a Gaussian charge distribution can be represented in an analytic form by Visscher and Dyall.^93^
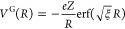
24which is the solution to the Poisson equation for the charge density defined by eq 22.

Among the different possibilities presented in ref (93), we have chosen the Gaussian model for the present work because it is simpler than the more realistic Fermi model and because it is implemented in all GTO codes. The goal of the present work is validation of the implementation. It is therefore less relevant which model is used provided that it is consistent throughout the software packages employed.

### Methods for Verification

To verify correctness of the ZORA implementation in MRChem, we use two other codes: a numerical atomic solver^84^ and an all-electron full-potential LAPW code EXCITING.^104^ In this section, we briefly introduce them and provide details on the implementation of new features needed for making a direct comparison with MRChem.

The atomic solver assumes atoms with spherically symmetric densities, and the monoelectronic wave functions are represented as , where  (*r*) is a radial function defined on a one-dimensional radial grid. The SCF of atomic solver uses an approach similar to that of the MW framework described above. It reduces the nonrelativistic Kohn–Sham equation to the integral form as follows
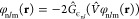
25

This equation matches eq 14, with vanishing off-diagonal terms of the Fock matrix because the canonical representation is used. The radial solver originally supported only nonrelativistic calculations: to implement ZORA, we used eq 17 in the canonical form, i.e., replacing *F*_*ii*_ with  and setting *F*_*ij*_ = 0 for *i* ≠ *j*. This approach avoids the evaluation of second derivatives and the corresponding numerical noise, which accumulates during the self-consistency iterations.

To consider systems beyond atoms, we use EXCITING code. In a nutshell, the code relies on partitioning the unit cell into nonoverlapping atomic spheres and the interstitial region. In the atomic spheres, the wave functions are expressed in terms of atomic-like orbitals that are updated during the self-consistence cycle. In the interstitial region, one represents the wave functions with plane waves using the smoothness of the Kohn–Sham potential. Based on such an approach, we use two types of basis functions: (i) augmented plane-waves (APWs)^95 ,96^ and (ii) local-orbitals (LOs).^97^ Each APW combines a plane wave in the interstitial region with atomic-like orbitals in the spheres, whereas each LO is a linear combination of two atomic-like orbitals in one particular sphere and strictly zero everywhere else. As shown in ref (86), it is possible to obtain a systematic convergence of the total energies simply by increasing the number of APWs and LOs.

The ZORA Hamiltonian is already available with the point nucleus model in the released version of EXCITING code in 2021, whereas the Gaussian nuclear model charge feature described in the Finite Nucleus Model section is implemented in the present work. Aside from adopting the electrostatic potential due to a Gaussian charge density, we also revise the evaluation of the following integral
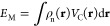
26

If a nucleus at the site **R**_0_ is defined as a point charge, then its density distribution is ρ_n_(**R**) = *Z*δ(**R** – **R**_0_) leading to *E*_M_ = *ZV*_C_(**R**_0_). For smeared nuclei with ρ_n_(**R**) = ρ^G^(**R**) (see eq 24), eq 26 is evaluated as an integral on a radial grid.

## Computational Details

All calculations were performed with the PBE functional^98^ using the XCFun^99^ library, the LibXC^100^ library, and the native implementation in MRChem, the atomic solver, and EXCITING, respectively. Being aware of slight inconsistencies in the PBE parameters employed in these libraries, we set β = 0.06672455060314922 and  as defined in XCFun and use these values in calculations with all three codes.

For all MW calculations, a development version of MRChem has been employed. Interpolating polynomials of ninth order were used with a simulation box size of 128 a_0_. The numerical precision thresholds used are summarized in Table 1.

**Table 1:** MRChem Precision Parameters

parameter	value	explanation
world_prec	1.0 × 10^–6^	overall numerical precision
energy_thrs	1.0 × 10^–6^	convergence threshold total energy
orbital_thrs	1.0 × 10^–4^	convergence threshold maximum orbital residual

All EXCITING calculations were performed using a large cubic unit cell with a side length of 25 a_0_ for atoms and 30 a_0_ for molecules. The electrostatic interaction of the periodic images was eliminated by introducing the truncation of the Coulombic interaction following the approach explained in ref (81). Aside from acquiring the isolated limit, this adjustment allows us to obtain ZORA energies consistent with both MRChem and the atomic solver. The unmodified electrostatic potential for periodic densities is defined uniquely except for an additive constant which introduces an ambiguity in the ZORA energies.^34,37,54−56^ The truncation of the Coulombic interaction makes the potential unique and thus removes this ambiguity completely.^34,37,54−56^ The canonical orbitals were expressed in terms of local orbitals and LAPWs with the cutoff *R*_MT_*K*_max_ sufficient to ensure a few μHa precision. The specific settings in the case of each atom and molecule are stored in Table 2, and a set of input and output data files are available in the repository.

**Table 2:** LAPW Parameters and Structural Data Used in the Calculations of the Considered Molecules and Atoms^a^

material	*R*_MT_^min^*G*_max_	*R*_MT_ [a_0_]	bond length [Å]
CaO	11	1.63/1.40	1.8221^101^
CuH	10	1.40/1.00	1.4626^101^
SrO	13	1.63/1.40	1.9050^102^
Cu_2_	14	1.8	2.2197^101^
AgH	11	1.68/1.20	1.6180^101^
I_2_	14	1.6	2.6630^102^
He	11	2	
Ne	12	2	
Ar	13	2	
Kr	13	2	
Xe	15	2	

a *R*_MT_^min^*G*_max_ is the product of the smallest muffin-tin radius and the largest reciprocal lattice vector.

The calculations with the atomic solver employed a fifth order polynomial on a radial grid with the innermost and outermost points *r*_min_ = 10^–8^ a_0_ and *r*_max_ = 35 a_0_. The number of radial points was set to 5000, which is fully sufficient to guarantee sub-μHa precision.

The total energies of diatomic molecules were calculated in MRChem and EXCITING without geometry relaxation using the internuclear distances given in Table 2. The bond lengths in Table 2 are taken from the NIST^101^ database with the exception of SrO and I_2_, which are private communication from not published work.^102^

## Results and Discussion

### Relative Contributions of the ZORA Terms

**Figure 1:**
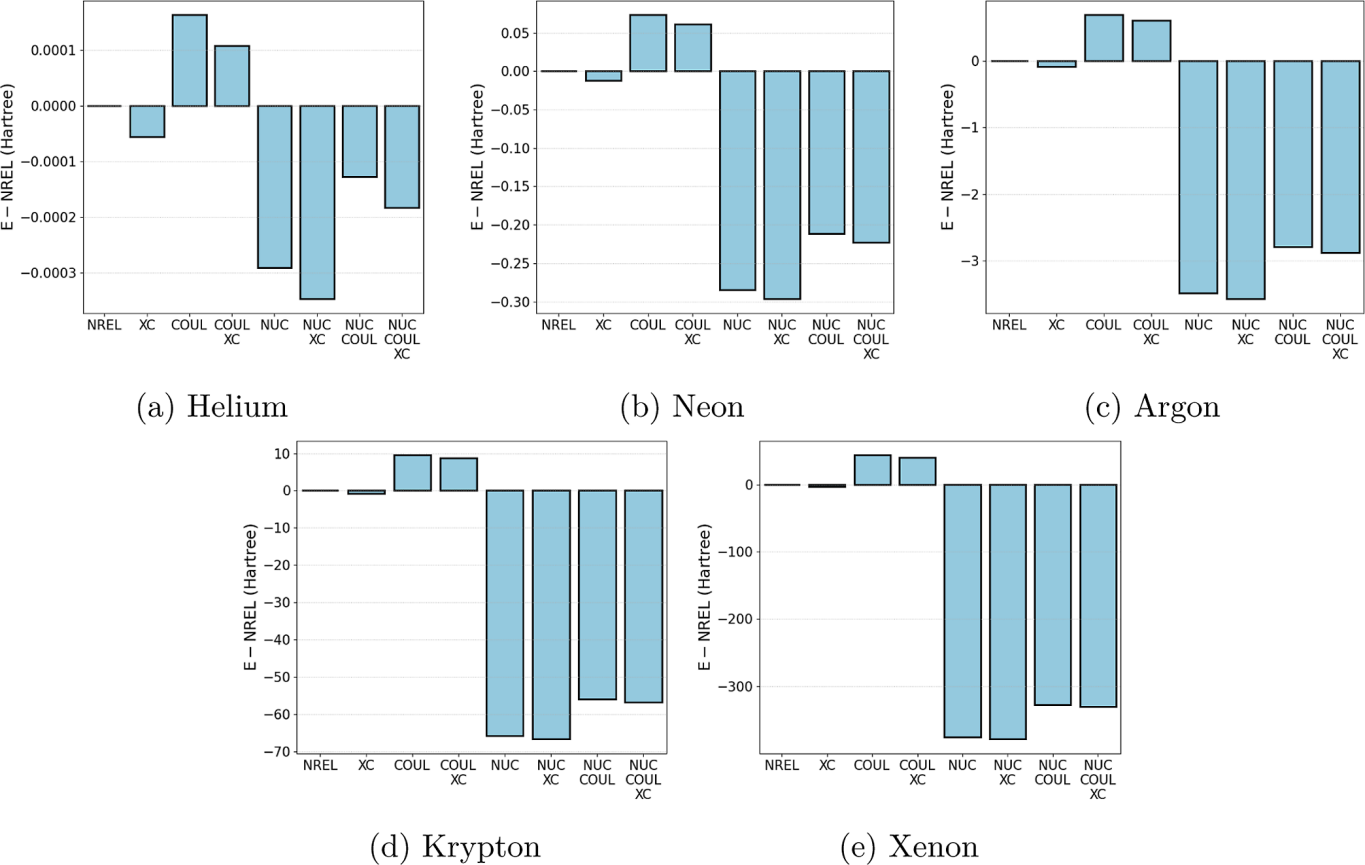
Relative contributions to the total energy (in Hartree) for all seven possible ZORA operators, compared to the nonrelativistic total energy, for the noble gases He–Xe. Note that the scales on the *y*-axes are different for each subplot.

The current MRChem implementation is able to include all local contributions of the potential into . It is therefore possible to measure their relative weights for a given atom and how their contribution changes with nuclear charge. We have performed a series of calculations for the noble gases from helium to xenon, with all seven possibilities: one contribution only, two contributions, and all three. The results are summarized in Figure 1. The nuclear potential  is the largest contribution, as expected. It is followed by the Coulombic term *Ĵ*, and the exchange and correlation potential  is the smallest one. Moreover, the relativistic correction increases roughly with the fourth power of the nuclear charge as expected,^5^ and the nuclear term becomes progressively more dominant for heavier atoms.

### Validation

To validate the ZORA implementation in MRChem, we perform total energy calculations of noble gas atoms and a small set of molecules broadly covering the first five rows of the periodic table (H–Xe). In the case of the atoms, we apply three different types of calculations: the atomic solver, LAPWs, and MWs. For the diatomic molecules, we compare the results obtained using MW and LAPW by means of MRChem and EXCITING, respectively.

To assess the potential agreement which can be achieved between the various codes, we have performed nonrelativistic calculations with all three codes, using point-charge nuclei (numerically smoothed as described in Point Nucleus Model section in the case of MWs). The results are summarized in Table 3. We have then obtained a root-mean-square deviation (rmsd) of the relative error between MWs and LAPWs equal to 5.21 × 10^–8^ and between MWs and atomic solver equal to 8.07 × 10^–10^. We concluded that the three methods are in very good agreement, setting the mark for what can be expected in the ZORA domain, using a Gaussian nuclear charge distribution as in eq 24. The ZORA results are summarized in Table 4, showing a rmsd for the relative errors between MWs and LAPWs equal to 3.95 × 10^–8^, and between MWs and atomic solver equal to 6.93 × 10^–9^, in line with what has been here previously observed for the nonrelativistic regime, thus confirming the validity of the implementation. In terms of absolute errors, we find that most discrepancies in the total energies are within 10 microHartrees, and only in two cases (AgH and I_2_) the differences are larger, yet they do not exceed 21 microHartrees. This level of agreement is much better than the so-called *chemical accuracy threshold* (1 kcal/mol).

**Table 3:** Non-Relativistic Total Energies (Given in Hartrees) Obtained Using Three Different Codes and Their Relative Differences^a^

				relative difference	
species	atomic solver	EXCITING	MRChem	MRChem vs atomic solver	MRChem vs EXCITING
He	–2.892935	–2.892935	–2.892935	0.0	0.0
Ne	–128.866434	–128.866433	–128.866433	–3.1 × 10^–^^10^	0.0
Ar	–527.346141	–527.346138	–527.346140	–1.7 × 10^–^^9^	3.7 × 10^–^^9^
Kr	–2753.416138	–2753.416137	–2753.416138	0.0	2.1 × 10^–^^10^
Xe	–7234.233259	–7234.233259	–7234.233259	0.0	0.0
CaO		–752.562050	–752.562058		1.0 × 10^–^^8^
CuH		–1640.901717	–1640.901727		6.1 × 10^–^^9^
SrO		–3208.161712	–3208.161722		3.1 × 10^–^^9^
Cu_2_		–3280.675840	–3280.675846		1.7 × 10^–^^9^
AgH		–5200.162245	–5200.162259		2.8 × 10^–^^9^
I_2_		–13840.158307	–13840.158328		1.5 × 10^–^^9^

a In all cases, the point-like nucleus model is used.

**Table 4:** Scalar-Relativistic ZORA Total Energies (Given in Hartrees) Obtained Using Three Different Codes and Their Relative Differences^a^

				relative difference	
species	atomic solver	EXCITING	MRChem	MRChem vs atomic solver	MRChem vs EXCITING
He	–2.893119	–2.893119	–2.893119	0.0	0.0
Ne	–129.089335	–129.089335	–129.089335	0.0	0.0
Ar	–530.224039	–530.224036	–530.224038	–2.6 × 10^–^^9^	2.9 × 10^–^^9^
Kr	–2810.049764	–2810.049762	–2810.049755	–3.1 × 10^–^^9^	–2.4 × 10^–^^9^
Xe	–7564.596665	–7564.596662	–7564.596554	–1.5 × 10^–^^8^	–1.4 × 10^–^^8^
CaO		–757.195492	–757.195491		–1.7 × 10^–^^9^
CuH		–1663.238493	–1663.238504		6.7 × 10^–^^9^
SrO		–3279.826587	–3279.826596		2.7 × 10^–^^9^
Cu_2_		–3325.342851	–3325.342843		–2.4 × 10^–^^9^
AgH		–5380.042000	–5380.042003		5.5 × 10^–^^10^
I_2_		–14448.747787	–14448.747761		–1.8 × 10^–^^9^

a In all cases, the smeared nucleus model is used.

Finally, we find that introducing the relativistic corrections in the MW formalism, as expressed in eq 17, leads to a minor increase in MRChem runtimes. Therefore, we anticipate that the analysis of the performance (runtimes and parallel scaling) given in ref (103) remains valid in the ZORA case.

## Conclusions

We have formulated the ZORA method in a form compatible with MWs and implemented it in the MRChem program. The validity and precision of the implementation have been tested against a radial, numerical atomic code and a plane wave code, showing excellent agreement. The current study was performed with the specific idea to validate method and theory with a small benchmark: atoms and diatomics, covering broadly the periodic table up to and including fifth-row elements. The model is also capable of dealing with all parts of the electronic potential self-consistently, with the exception of the HF exchange, which is the only one that cannot be expressed in the closed form.

## References

[ref1] Dirac P. A. M. Theory of Electrons and Positrons, Nobel Lecture; Scientific Research Publishing Inc. 1933; pp 320–325.

[ref2] Dirac P. A. M. The Quantum Theory of the Electron. Proc. R. Soc. Lond. - Ser. A Contain. Pap. a Math. Phys. Character 1928, 117, 610–624.

[ref3] Simões A. Dirac’s Claim and the Chemists. Phys. Perspect. 2002, 4, 253–266. 10.1007/s00016-002-8369-1.

[ref4] Bartlett N. Relativistic effects and the chemistry of gold. Gold Bull. 1998, 31, 22–25. 10.1007/BF03215471.

[ref5] Pyykkö P. Relativistic Effects in Chemistry: More Common Than You Thought. Annu. Rev. Phys. Chem. 2012, 63, 45–64. 10.1146/annurev-physchem-032511-143755. 22404585

[ref6] Keeton S.; Loucks T. Electronic structure of mercury. Phys. Rev. 1966, 152, 548–555. 10.1103/PhysRev.152.548.

[ref7] Ahuja R.; Blomqvist A.; Larsson P.; Pyykkö P.; Zaleski-Ejgierd P. Relativity and the lead-acid battery. Phys. Rev. Lett. 2011, 106, 018301 10.1103/PhysRevLett.106.018301. 21231773

[ref8] Demissie T. B.; Garabato B. D.; Ruud K.; Kozlowski P. M. Mercury Methylation by Cobalt Corrinoids: Relativistic Effects Dictate the Reaction Mechanism. Angew. Chem., Int. Ed. 2016, 55, 11503–11506. 10.1002/anie.201606001. 27510509

[ref9] Vícha J.; Marek R.; Straka M. High-Frequency 13C and 29Si NMR Chemical Shifts in Diamagnetic Low-Valence Compounds of TlI and PbII: Decisive Role of Relativistic Effects. Inorg. Chem. 2016, 55, 1770–1781. 10.1021/acs.inorgchem.5b02689. 26820039

[ref10] Lein M.; Rudolph M.; Hashmi S. K.; Schwerdtfeger P. Homogeneous Gold Catalysis: Mechanism and Relativistic Effects of the Addition of Water to Propyne. Organometallics 2010, 29, 2206–2210. 10.1021/om900710v.

[ref11] Boualili F. Z.; Nemouchi M.; Godefroid M.; Jönsson P. Weak correlation and strong relativistic effects on the hyperfine interaction in fluorine. Phys. Rev. A 2021, 104, 062813 10.1103/PhysRevA.104.062813.

[ref12] Mattsson S.; Paulus B.; Redeker F. A.; Beckers H.; Riedel S.; Müller C. The Crystal Structure of α-F2: Solving a 50 Year Old Puzzle Computationally. Chem.--Eur. J. 2019, 25, 3318–3324. 10.1002/chem.201805300. 30620433

[ref13] Tantardini C.; Jalolov F. N.; Kvashnin A. G. Crystal Structure Evolution of Fluorine under High Pressure. J. Phys. Chem. C 2022, 126, 11358–11364. 10.1021/acs.jpcc.2c02213.

[ref14] Hellmann H. A new approximation method in the problem of many electrons. J. Chem. Phys. 1935, 3, 61–62. 10.1063/1.1749559.

[ref15] Gombás P. Über die metallische Bindung. Z. Phys. 1935, 94, 473–488. 10.1007/BF01330613.

[ref16] Xu X.; Truhlar D. G. Accuracy of Effective Core Potentials and Basis Sets for Density Functional Calculations, Including Relativistic Effects, As Illustrated by Calculations on Arsenic Compounds. J. Chem. Theory Comput. 2011, 7, 2766–2779. 10.1021/ct200234r. 26605468

[ref17] Stoll H.; Metz B.; Dolg M. Relativistic energy-consistent pseudopotentials-Recent developments. J. Comput. Chem. 2002, 23, 767–778. 10.1002/jcc.10037. 12012353

[ref18] Heine V. The Pseudopotential Concept. In Solid State Physics; Ehrenreich H., Seitz F., Turnbull D., Eds.; Academic Press, 1970; Vol. 24, pp 1–36.

[ref19] Saue T. Relativistic Hamiltonians for chemistry: A primer. ChemPhysChem 2011, 12, 3077–3094. 10.1002/cphc.201100682. 22076930

[ref20] Rudek B.; Toyota K.; Foucar L.; Erk B.; Boll R.; Bomme C.; Correa J.; Carron S.; Boutet S.; Williams G. J.; et al. Relativistic and resonant effects in the ionization of heavy atoms by ultra-intense hard X-rays. Nat. Commun. 2018, 9, 4200 10.1038/s41467-018-06745-6. 30305630 PMC6180123

[ref21] Breit G. An Interpretation of Dirac’s Theory of the Electron. Proc. Natl. Acad. Sci. U.S.A. 1928, 14, 553–559. 10.1073/pnas.14.7.553. 16587362 PMC1085609

[ref22] Breit G. Dirac’s Equation and the Spin-Spin Interactions of Two Electrons. Phys. Rev. 1932, 39, 616–624. 10.1103/PhysRev.39.616.

[ref23] Moss R. Advanced Molecular Quantum Mechanics: An Introduction to Relativistic Quantum Mechanics and the Quantum Theory of Radiation; Springer Science & Business Media, 2012.

[ref24] Dyall K. G.; Fægri K. Introduction to Relativistic Quantum Chemistry; Oxford University Press, 2007.

[ref25] Helgaker T.; Coriani S.; Jørgensen P.; Kristensen K.; Olsen J.; Ruud K. Recent Advances in Wave Function-Based Methods of Molecular-Property Calculations. Chem. Rev. 2012, 112, 543–631. 10.1021/cr2002239. 22236047

[ref26] Jacob C. R.; Reiher M. Spin in density-functional theory. Int. J. Quantum Chem. 2012, 112, 3661–3684. 10.1002/qua.24309.

[ref27] Marian C. M. Spin–orbit coupling and intersystem crossing in molecules. Wiley Interdiscip. Rev.: Comput. Mol. Sci. 2012, 2, 187–203. 10.1002/wcms.83.

[ref28] Repisky M.; Komorovsky S.; Kadek M.; Konecny L.; Ekström U.; Malkin E.; Kaupp M.; Ruud K.; Malkina O. L.; Malkin V. G. ReSpect: Relativistic spectroscopy DFT program package. J. Chem. Phys. 2020, 152, 184101 10.1063/5.0005094. 32414255

[ref29] Saue T.; Bast R.; Gomes A. S. P.; Jensen H. J. A.; Visscher L.; Aucar I. A.; Di Remigio R.; Dyall K. G.; Eliav E.; Fasshauer E.; et al. The DIRAC code for relativistic molecular calculations. J. Chem. Phys. 2020, 152, 204104 10.1063/5.0004844. 32486677

[ref30] Zhang Y.; Suo B.; Wang Z.; Zhang N.; Li Z.; Lei Y.; Zou W.; Gao J.; Peng D.; Pu Z.; et al. BDF: A relativistic electronic structure program package. J. Chem. Phys. 2020, 152, 064113 10.1063/1.5143173. 32061235

[ref31] Belpassi L.; De Santis M.; Quiney H. M.; Tarantelli F.; Storchi L. BERTHA: Implementation of a four-component Dirac–Kohn–Sham relativistic framework. J. Chem. Phys. 2020, 152, 164118 10.1063/5.0002831. 32357778

[ref32] Foldy L. L.; Wouthuysen S. A. On the Dirac Theory of Spin 1/2 Particles and Its Non-Relativistic Limit. Phys. Rev. 1950, 78, 29–36. 10.1103/PhysRev.78.29.

[ref33] Foldy L. L. The Electromagnetic Properties of Dirac Particles. Phys. Rev. 1952, 87, 688–693. 10.1103/PhysRev.87.688.

[ref34] Chang C.; Pelissier M.; Durand P. Regular two-component Pauli-like effective Hamiltonians in Dirac theory. Phys. Scr. 1986, 34, 394–404. 10.1088/0031-8949/34/5/007.

[ref35] Kang H.; Uhlmann G. Inverse problems for the Pauli Hamiltonian in two dimensions. J. Fourier Anal. Appl. 2004, 10, 201–215. 10.1007/s00041-004-8011-5.

[ref36] Goldman T. Gauge invariance, time-dependent Foldy-Wouthuysen transformations, and the Pauli Hamiltonian. Phys. Rev. D 1977, 15, 1063–1067. 10.1103/PhysRevD.15.1063.

[ref37] van Lenthe E.; Baerends E.-J.; Snijders J. G. Relativistic total energy using regular approximations. J. Chem. Phys. 1994, 101, 9783–9792. 10.1063/1.467943.

[ref38] Dyall K. G.; van Lenthe E. Relativistic regular approximations revisited: An infinite-order relativistic approximation. J. Chem. Phys. 1999, 111, 1366–1372. 10.1063/1.479395.

[ref39] Mohri M.; Nederhof M.-J. Robustness in Language and Speech Technology; Springer, 2001; pp 153–163.

[ref40] Douglas M.; Kroll N. M. Quantum electrodynamical corrections to the fine structure of helium. Ann. Phys. 1974, 82, 89–155. 10.1016/0003-4916(74)90333-9.

[ref41] Hess B. A. Applicability of the no-pair equation with free-particle projection operators to atomic and molecular structure calculations. Phys. Rev. A 1985, 32, 756–763. 10.1103/PhysRevA.32.756. 9896123

[ref42] Hess B. A. Relativistic electronic-structure calculations employing a two-component no-pair formalism with external-field projection operators. Phys. Rev. A 1986, 33, 3742–3748. 10.1103/PhysRevA.33.3742. 9897114

[ref43] Jansen G.; Heß B. A. Revision of the Douglas-Kroll transformation. Phys. Rev. A 1989, 39, 6016–6017. 10.1103/PhysRevA.39.6016. 9901188

[ref44] Reiher M. Douglas–Kroll–Hess Theory: a relativistic electrons-only theory for chemistry. Theor. Chem. Acc. 2006, 116, 241–252. 10.1007/s00214-005-0003-2.

[ref45] Kutzelnigg W.; Liu W. Quasirelativistic theory equivalent to fully relativistic theory. J. Chem. Phys. 2005, 123, 241102 10.1063/1.2137315. 16396527

[ref46] Liu W.; Peng D. Infinite-order quasirelativistic density functional method based on the exact matrix quasirelativistic theory. J. Chem. Phys. 2006, 125, 044102 10.1063/1.2222365. 16942129

[ref47] Liu W.; Peng D. Exact two-component Hamiltonians revisited. J. Chem. Phys. 2009, 131, 031104 10.1063/1.3159445. 19624172

[ref48] Iliaš M.; Saue T. An infinite-order two-component relativistic Hamiltonian by a simple one-step transformation. J. Chem. Phys. 2007, 126, 064102 10.1063/1.2436882. 17313208

[ref49] Peng D.; Liu W.; Xiao Y.; Cheng L. Making four-and two-component relativistic density functional methods fully equivalent based on the idea of “from atoms to molecule. J. Chem. Phys. 2007, 127, 104106 10.1063/1.2772856. 17867736

[ref50] Peng D.; Middendorf N.; Weigend F.; Reiher M. An efficient implementation of two-component relativistic exact-decoupling methods for large molecules. J. Chem. Phys. 2013, 138, 184105 10.1063/1.4803693. 23676027

[ref51] Liu W. Ideas of relativistic quantum chemistry. Mol. Phys. 2010, 108, 1679–1706. 10.1080/00268971003781571.

[ref52] Liu W. Advances in relativistic molecular quantum mechanics. Phys. Rep. 2014, 537, 59–89. 10.1016/j.physrep.2013.11.006.

[ref53] Liu W. Big picture of relativistic molecular quantum mechanics. Natl. Sci. Rev. 2016, 3, 204–221. 10.1093/nsr/nwv081.

[ref54] Filatov M.; Cremer D. On the physical meaning of the ZORA Hamiltonian. Mol. Phys. 2003, 101, 2295–2302. 10.1080/0026897031000137670.

[ref55] Heully J.-L.; Lindgren I.; Lindroth E.; Lundqvist S.; Martensson-Pendrill A.-M. Diagonalisation of the Dirac Hamiltonian as a basis for a relativistic many-body procedure. J. Phys. B Atom. Mol. Phys. 1986, 19, 2799–2815. 10.1088/0022-3700/19/18/011.

[ref56] Lenthe E. v.; Baerends E.-J.; Snijders J. G. Relativistic regular two-component Hamiltonians. J. Chem. Phys. 1993, 99, 4597–4610. 10.1063/1.466059.

[ref57] Pantazis D. A.; Neese F. All-electron basis sets for heavy elements. Wiley Interdiscip. Rev. Comput. Mol. Sci. 2014, 4, 363–374. 10.1002/wcms.1177.

[ref58] Güell M.; Luis J. M.; Sola M.; Swart M. Importance of the basis set for the spin-state energetics of iron complexes. J. Phys. Chem. A 2008, 112, 6384–6391. 10.1021/jp803441m. 18572904

[ref59] van Leeuwen R.; van Lenthe E.; Baerends E. J.; Snijders J. G. Exact solutions of regular approximate relativistic wave equations for hydrogen-like atoms. J. Chem. Phys. 1994, 101, 1272–1281. 10.1063/1.467819.

[ref60] de Castro E. V. R.; Jorge F. E. Accurate universal Gaussian basis set for all atoms of the Periodic Table. J. Chem. Phys. 1998, 108, 5225–5229. 10.1063/1.475959.

[ref61] Zobel J. P.; Widmark P.-O.; Veryazov V. The ANO-R Basis Set. J. Chem. Theory Comput. 2020, 16, 278–294. 10.1021/acs.jctc.9b00873. 31738554

[ref62] Zobel J. P.; Widmark P.-O.; Veryazov V. Correction to “The ANO-R Basis Set. J. Chem. Theory Comput. 2021, 17, 3233–3234. 10.1021/acs.jctc.1c00329. 33877850

[ref63] Roos B.; Veryazov V.; Widmark P.-O. Relativistic atomic natural orbital type basis sets for the alkaline and alkaline-earth atoms applied to the ground-state potentials for the corresponding dimers. Theor. Chem. Acc. 2004, 111, 345–351. 10.1007/s00214-003-0537-0.

[ref64] Roos B. O.; Lindh R.; Malmqvist P. Å.; Veryazov V.; Widmark P. O. Main Group Atoms and Dimers Studied with a New Relativistic ANO Basis Set. J. Phys. Chem. A 2004, 108, 2851–2858. 10.1021/jp031064+.

[ref65] Roos B. O.; Lindh R.; Malmqvist P. Å.; Veryazov V.; Widmark P. O. New Relativistic ANO Basis Sets for Transition Metal Atoms. J. Phys. Chem. A 2005, 109, 6575–6579. 10.1021/jp0581126. 16834004

[ref66] Pollak P.; Weigend F. Segmented Contracted Error-Consistent Basis Sets of Double- and Triple-ζ Valence Quality for One- and Two-Component Relativistic All-Electron Calculations. J. Chem. Theory Comput. 2017, 13, 3696–3705. 10.1021/acs.jctc.7b00593. 28679044

[ref67] Pantazis D. A.; Neese F. All-electron scalar relativistic basis sets for the lanthanides. J. Chem. Theory Comput. 2009, 5, 2229–2238. 10.1021/ct900090f. 26616609

[ref68] Pantazis D. A.; Neese F. All-electron scalar relativistic basis sets for the actinides. J. Chem. Theory Comput. 2011, 7, 677–684. 10.1021/ct100736b. 26616609

[ref69] Pantazis D. A.; Neese F. All-electron scalar relativistic basis sets for the 6 p elements. Theor. Chem. Acc. 2012, 131, 1292–1297. 10.1007/s00214-012-1292-x.

[ref70] Aravena D.; Neese F.; Pantazis D. A. Improved segmented all-electron relativistically contracted basis sets for the lanthanides. J. Chem. Theory Comput. 2016, 12, 1148–1156. 10.1021/acs.jctc.5b01048. 26839966

[ref71] Rolfes J. D.; Neese F.; Pantazis D. A. All-electron scalar relativistic basis sets for the elements Rb–Xe. J. Comput. Chem. 2020, 41, 1842–1849. 10.1002/jcc.26355. 32484577

[ref72] Alpert B. K. A Class of Bases in L^2^ for the Sparse Representation of Integral Operators. SIAM J. Math. Anal. 1993, 24, 246–262. 10.1137/0524016.

[ref73] Jensen S. R.; Saha S.; Flores-Livas J. A.; Huhn W.; Blum V.; Goedecker S.; Frediani L. The elephant in the room of density functional theory calculations. J. Phys. Chem. Lett. 2017, 8, 1449–1457. 10.1021/acs.jpclett.7b00255. 28291362

[ref74] Keinert F. Encyclopedia of Complexity and Systems Science; Meyers R. A., Ed.; Springer New York, 2009; pp 5841–5858.

[ref75] Harrison R. J.; Fann G. I.; Yanai T.; Gan Z.; Beylkin G. Multiresolution quantum chemistry: Basic theory and initial applications. J. Chem. Phys. 2004, 121, 11587–11598. 10.1063/1.1791051. 15634124

[ref76] Kato T.; Yokoi Y.; Sekino H. Basis set limit computation of dynamic polarizability at near-resonance region. Int. J. Quantum Chem. 2013, 113, 286–289. 10.1002/qua.24148.

[ref77] Jensen S. R.; Saha S.; Flores-Livas J. A.; Huhn W.; Blum V.; Goedecker S.; Frediani L. The Elephant in the Room of Density Functional Theory Calculations. J. Phys. Chem. Lett. 2017, 8, 1449–1457. 10.1021/acs.jpclett.7b00255. 28291362

[ref78] Alpert B.; Beylkin G.; Gines D.; Vozovoi L. Adaptive Solution of Partial Differential Equations in Multiwavelet Bases. J. Comput. Phys. 2002, 182, 149–190. 10.1006/jcph.2002.7160.

[ref79] Beylkin G.; Cheruvu V.; Pérez F. Fast adaptive algorithms in the non-standard form for multidimensional problems. Appl. Comput. Harmon. Anal. 2008, 24, 354–377. 10.1016/j.acha.2007.08.001.

[ref80] Frediani L.; Fossgaard E.; Flå T.; Ruud K. Fully adaptive algorithms for multivariate integral equations using the non-standard form and multiwavelets with applications to the Poisson and bound-state Helmholtz kernels in three dimensions. Mol. Phys. 2013, 111, 1143–1160. 10.1080/00268976.2013.810793.

[ref81] Jensen S. R.; Flå T.; Jonsson D.; Monstad R. S.; Ruud K.; Frediani L. Magnetic properties with multiwavelets and DFT: the complete basis set limit achieved. Phys. Chem. Chem. Phys. 2016, 18, 21145–21161. 10.1039/C6CP01294A. 27087397

[ref82] Bischoff F. A.; Harrison R. J.; Valeev E. F. Computing many-body wave functions with guaranteed precision: The first-order Møller-Plesset wave function for the ground state of helium atom. J. Chem. Phys. 2012, 137, 104103 10.1063/1.4747538. 22979846

[ref83] Anderson J.; Sundahl B.; Harrison R.; Beylkin G. Dirac-Fock calculations on molecules in an adaptive multiwavelet basis. J. Chem. Phys. 2019, 151, 234112 10.1063/1.5128908. 31864249

[ref84] Užulis J.; Gulans A. Radial Kohn–Sham problem via integral-equation approach. J. Phys. Commun. 2022, 6, 085002 10.1088/2399-6528/ac82a5.

[ref85] Gulans A.; Kontur S.; Meisenbichler C.; Nabok D.; Pavone P.; Rigamonti S.; Sagmeister S.; Werner U.; Draxl C. exciting: a full-potential all-electron package implementing density-functional theory and many-body perturbation theory. J. Phys.: Condens. Matter 2014, 26, 363202 10.1088/0953-8984/26/36/363202. 25135665

[ref86] Gulans A.; Kozhevnikov A.; Draxl C. Microhartree precision in density functional theory calculations. Phys. Rev. B 2018, 97, 161105 10.1103/PhysRevB.97.161105.

[ref87] Knuth F.; Carbogno C.; Atalla V.; Blum V.; Scheffler M. All-electron formalism for total energy strain derivatives and stress tensor components for numeric atom-centered orbitals. Comput. Phys. Commun. 2015, 190, 33–50. 10.1016/j.cpc.2015.01.003.

[ref88] Kalos M. H. Monte Carlo Calculations of the Ground State of Three- and Four-Body Nuclei. Phys. Rev. 1962, 128, 1791–1795. 10.1103/PhysRev.128.1791.

[ref89] Beylkin G.; Mohlenkamp M. J. Numerical operator calculus in higher dimensions. Proc. Natl. Acad. Sci. U.S.A. 2002, 99, 10246–10251. 10.1073/pnas.112329799. 12140360 PMC124898

[ref90] Faas S.; van Lenthe J. H.; Hennum A. C.; Snijders J. G. An ab initio two-component relativistic method including spin–orbit coupling using the regular approximation. J. Chem. Phys. 2000, 113, 4052–4059. 10.1063/1.1288387.

[ref91] Visscher L.; Saue T. Approximate relativistic electronic structure methods based on the quaternion modified Dirac equation. J. Chem. Phys. 2000, 113, 3996–4002. 10.1063/1.1288371.

[ref92] MRChem Program Package, 2020.

[ref93] Visscher L.; Dyall K. Dirac-Fock Atomic Electronic Structure Calculations Using Different Nuclear Charge Distributions. Atomic Data Nucl. Data Tables 1997, 67, 207–224. 10.1006/adnd.1997.0751.

[ref94] Johnson W.; Soff G. The lamb shift in hydrogen-like atoms, 1 - Z - 110. Atomic Data Nucl. Data Tables 1985, 33, 405–446. 10.1016/0092-640X(85)90010-5.

[ref95] Slater J. C. Wave functions in a periodic potential. Phys. Rev. 1937, 51, 846–851. 10.1103/PhysRev.51.846.

[ref96] Andersen O. K. Linear methods in band theory. Phys. Rev. B: Solid State 1975, 12, 3060–3083. 10.1103/PhysRevB.12.3060.

[ref97] Sjöstedt E.; Nordström L.; Singh D. J. An alternative way of linearizing the augmented plane-wave method. Solid State Commun. 2000, 114, 15–20. 10.1016/S0038-1098(99)00577-3.

[ref98] Perdew J. P.; Burke K.; Ernzerhof M. Generalized Gradient Approximation Made Simple. Phys. Rev. Lett. 1996, 77, 3865–3868. 10.1103/PhysRevLett.77.3865. 10062328

[ref99] Ekström U.; Visscher L.; Bast R.; Thorvaldsen A. J.; Ruud K. Arbitrary-Order Density Functional Response Theory from Automatic Differentiation. J. Chem. Theory Comput. 2010, 6, 1971–1980. 10.1021/ct100117s. 26615926

[ref100] Lehtola S.; Steigemann C.; Oliveira M. J.; Marques M. A. Recent developments in libxc-A comprehensive library of functionals for density functional theory. SoftwareX 2018, 7, 1–5. 10.1016/j.softx.2017.11.002.

[ref101] Huber K. P.; Herzberg G. H. Constants of Diatomic Molecules (Data Prepared by Jean W. Gallagher and Russell D. Johnson, III) in NIST Chemistry WebBook, NIST Standard Reference Database Number 69; Linstrom P. J., Mallard W. G., Eds.; National Institute of Standards and Technology, Gaithersburg MD; 2023, 20899, (retrieved November 24, 2023).

[ref102] Brakestad A. Private communication.

[ref103] Wind P.; Bjørgve M.; Brakestad A.; Gerez S G. A.; Jensen S. R.; Eikås R. D. R.; Frediani L. MRChem Multiresolution Analysis Code for Molecular Electronic Structure Calculations: Performance and Scaling Properties. J. Chem. Theory Comput. 2023, 19, 137–146. 10.1021/acs.jctc.2c00982. 36410396 PMC9835826

[ref104] Dewhurst J.; Sharma S.; Ambrosch-Draxl C.; Brouder C. The EXCITING Code Users’ Manual Version 0.9. 74.

